# Dentofacial and skeletal effects of two orthodontic maxillary protraction protocols: bone anchors versus facemask

**DOI:** 10.1186/s13005-024-00462-w

**Published:** 2024-10-18

**Authors:** Maike Tabellion, Jörg Alexander Lisson

**Affiliations:** Department of Orthodontics (G56), Saarland University, Kirrberger Strasse 100, 66424 Homburg, Saar Germany

**Keywords:** Maxillary retrognathia, Mandibular prognathia, Maxillary protraction, Bone anchors, Facemask

## Abstract

**Background:**

Maxillary retrognathia and/or mandibular prognathia are resulting in class III malocclusion. Regarding orthodontic class III malocclusion treatment, the literature reports several treatment approaches. This comparative clinical study investigated two maxillary protraction protocols including bone anchors and Delaire type facemask.

**Methods:**

Cephalometric radiographs of *n* = 31 patients were used for data acquisition. The patients were divided into two groups according to their treatment protocol: bone anchored protraction (*n* = 12, 8 female, 4 male; mean age 11.00 ± 1.76 years; average application: 13.50 ± 5.87 months) and facemask protraction (*n* = 19, 11 female, 8 male; mean age 6.74 ± 1.15 years; average application: 9.95 ± 4.17 months). The evaluation included established procedures for measurements of the maxilla, mandibula, incisor inclination and soft tissue. Statistics included Shapiro-Wilk- and T-Tests for the radiographs. The level of significance was set at *p* < 0.05.

**Results:**

The cephalometric analysis showed differences among the two groups. SNA angle showed significant improvements during protraction with bone anchors (2.30 ± 1.18°) with increase in the Wits appraisal of 2.01 ± 2.65 mm. SNA angle improved also during protraction with facemask (1.22 ± 2.28°) with increase in the Wits appraisal of 1.85 ± 4.09 mm. Proclination of maxillary incisors was larger in patients with facemask (3.35 ± 6.18°) and ML-SN angle increased more (1.05 ± 1.51°) than in patients with bone anchors. Loosening rate of bone anchors was 14.58%.

**Conclusions:**

Both treatment protocols led to correction of a class III malocclusion. However, this study was obtained immediately after protraction treatment and longitudinal observations after growth spurt will be needed to verify the treatment effects over a longer period. The use of skeletal anchorage for maxillary protraction reduces unwanted side effects and increases skeletal effects needed for class III correction.

## Introduction

Class III malocclusion comprises a variety of skeletal and dentoalveolar anomalies coming along with maxillary retrognathia or eugnathia combined with mandibular prognathia or eugnathia [8, 11, 14]. Each patient presents an individual class III malocclusion with different response to treatment approaches. The quality of maxillary protraction treatment depends on the method that is used. Extraoral maxillary protraction using a facemask has been the possibility to advance the maxilla improving growth during deciduous or early mixed dentition for ages. Successful advancement of the maxilla has been shown [2, 7]. However, unwanted side effects of facemask protraction have arisen [2, 4, 11]. Studies of class III treatment effects using the facemask described in fact skeletal maxillary protraction, but also proclination of maxillary incisors, set back of the chin with increasing lower face height due to clockwise rotation of the mandibula, extrusion of upper molars and retroclination of mandibular incisors depending on age of the patient, force, direction and duration of maxillary protraction [26]. Infrequent use because of extraoral anchorage and therefore less integration to daily routine of the patients led to different treatment outcome [21, 31]. To minimize the unwanted side effects and for better integration to daily routine, skeletal anchorage has been developed as a new treatment method. Patients have been treated with bone anchors attached to maxillary bone in the molar region and mandibular bone in the incisor region combined with class III elastics engaged to the bone anchors [7]. This approach was first introduced by De Clerk et al. [10]. Unwanted skeletal and dental effects have been reduced and a low failure rate has been described. Stimulation and modification of maxillary growth must be done at an early age. Otherwise, if the patient is advanced in age and growth is completed, orthognathic surgery is the only possibility to change maxillary and/or mandibular position [21]. Orthopedic treatment may reduce the necessity of orthognathic surgery or at least decrease the extent of that treatment [7]. Correcting the sagittal development of the maxilla by postero-anterior traction can only be done because of its forward displacement, compensatory reaction of the sutures, apposition-resorption processes and development in the anterior and lateral regions. Especially sutures articulating with the frontal, zygomatic, ethmoid and palatal bones effect maxillary growth. It is believed, that during facial growth, the cartilaginous nasal septum is the primary force in pacing morphogenesis of the maxilla and the surrounding bones. Activity of the suture and expansion of cartilaginous nasal septum ceases after about seven years of age. After that apposition processes over all surfaces are the prevalent growth mechanism [16]. Furthermore, tongue pressure against the palatal vault leads to growth of the maxilla in anterior and lateral regions. Growth of the maxilla takes place at its articular and posterior margins thrusting in a downward and forward direction [2, 12, 14, 16]. Since the growth pattern is unpredictable, it is difficult to forecast which patient with class III malocclusion can be treated successfully by orthopedic appliances alone or whether orthognathic surgery is required [21]. To our knowledge, the literature lacks studies that evaluate especially the effects of the bone anchored maxillary protraction protocol. It was introduced by De Clerk et al. [11] and comprises four titanium miniplates attached to the infrazygomatic crests and between the canine and lateral incisor of the mandibula on both sides. The literature reports several treatment approaches regarding class III malocclusion treatment with bone anchorage, such as hybrid hyrax in the maxilla and mentoplates in the mandible or orthodontic mini-implants instead of titanium miniplates [20]. This study adds value to the current literature by means of comparing different treatment methodologies for maxillary protraction particularly regarding bone anchorage using titanium miniplates in upper and lower jaws.

### Aims of the study

Since many orthopedic treatment protocols regarding class III malocclusion coexist, this study investigated two maxillary protraction protocols with and without skeletal anchorage in growing patients. The purpose was to evaluate skeletal, dentolaveolar and soft-tissue treatment effects in patients with bone anchors, compared to patients with a tooth-borne facemask. Feasible complications in conjunction with bone anchors should be considered.

## Methods

### Patients

The patients were divided into two groups depending on treatment protocol (bone anchors and facemask) with respect to age and compared to each other. Cephalometric radiographs of 31 non-syndromic patients (12 bone anchors, 19 facemask) at the age of 11.00 ± 1.76 years (bone anchors) and 6.74 ± 1.15 years (facemask) were identified and analyzed. All patients were exclusively diagnosed for orthodontic treatment at Saarland University Hospital. The treated sample of patients with bone anchors was collected prospectively over a period of four years now, since bone anchored maxillary protraction treatment was first introduced in our clinic at the end of 2019. The treated sample of patients with facemask was collected mainly prospectively between 2019 and 2024, but partially retrospectively between 2014 and 2018 to increase the number of participants.

### Inclusion/Exclusion criteria

The presence of maxillary retrognathia (SNA angle < 79°) and/or mandibular prognathia (SNB angle > 81°) and Wits appraisal of ≤ 0.0 mm were the inclusion criteria for both groups. The limit for SNA angle for maxillary eugnathia was set at 79° to 83°. The limit for SNB angle for mandibular eugnathia was set at 77° to 81° [17]. Exclusion criteria included comorbid syndromes and genetic disorders.

As a precondition, diagnostic data including digital cephalometric radiographs had to be present. Data were extracted from before the beginning of orthodontic treatment and at the end of maxillary protraction treatment.

### Control group

The patients with bone anchors (*n* = 12) were compared to patients with facemask (*n* = 19).

Since treatment with bone anchors was first introduced in our clinic in 2019 with two to four patients per year agreeing with this treatment, sample size determination was only partially possible. Against this background, we collected *n* = 20 patients in the bone anchors group for our investigation. Since our surgeons changed the bone anchor system at the beginning of 2024, the sample size matching the inclusion criteria until then was *n* = 12. *N* = 8 patients were excluded from the study, since *n* = 3 patients showed poor compliance and ended the treatment ahead of time and *n* = 5 did not want to undergo the surgical procedure and declined the treatment. Between 2019 and 2024 we did not have the same number of patients with facemask, therefore, we investigated existing diagnostic data of patients with facemask treatment back until 2014. Out of *n* = 20 patients with facemask, *n* = 19 patients met inclusion criteria, *n* = 1 patient was excluded. Both groups did not receive prior orthodontic treatment. None of the patients showed agenesis of permanent incisors.

### Treatment protocol of patients with bone anchors

**Fig. 1:**
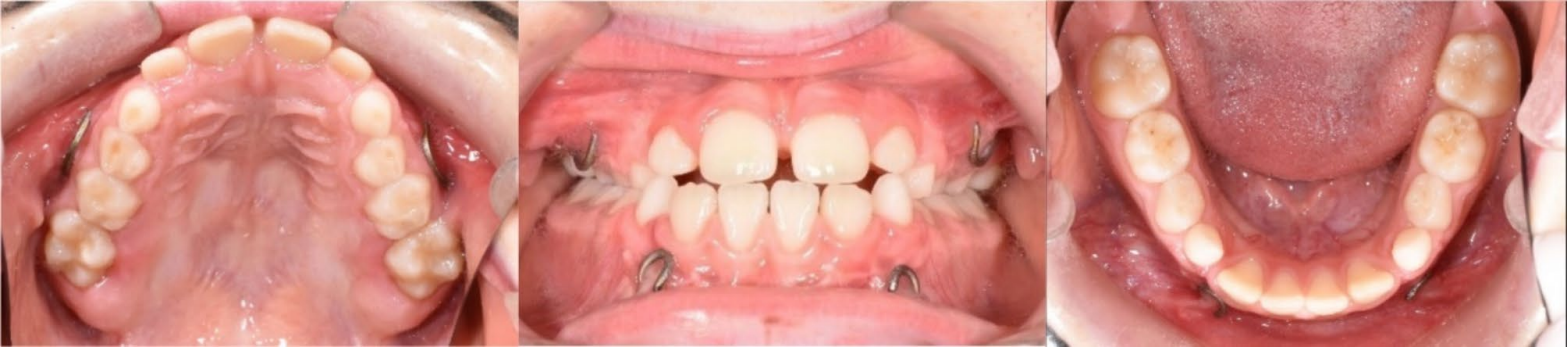
Pretreatment intraoral photographs after placement of bone anchors in upper and lower jaw on both sides

**Fig. 2:**
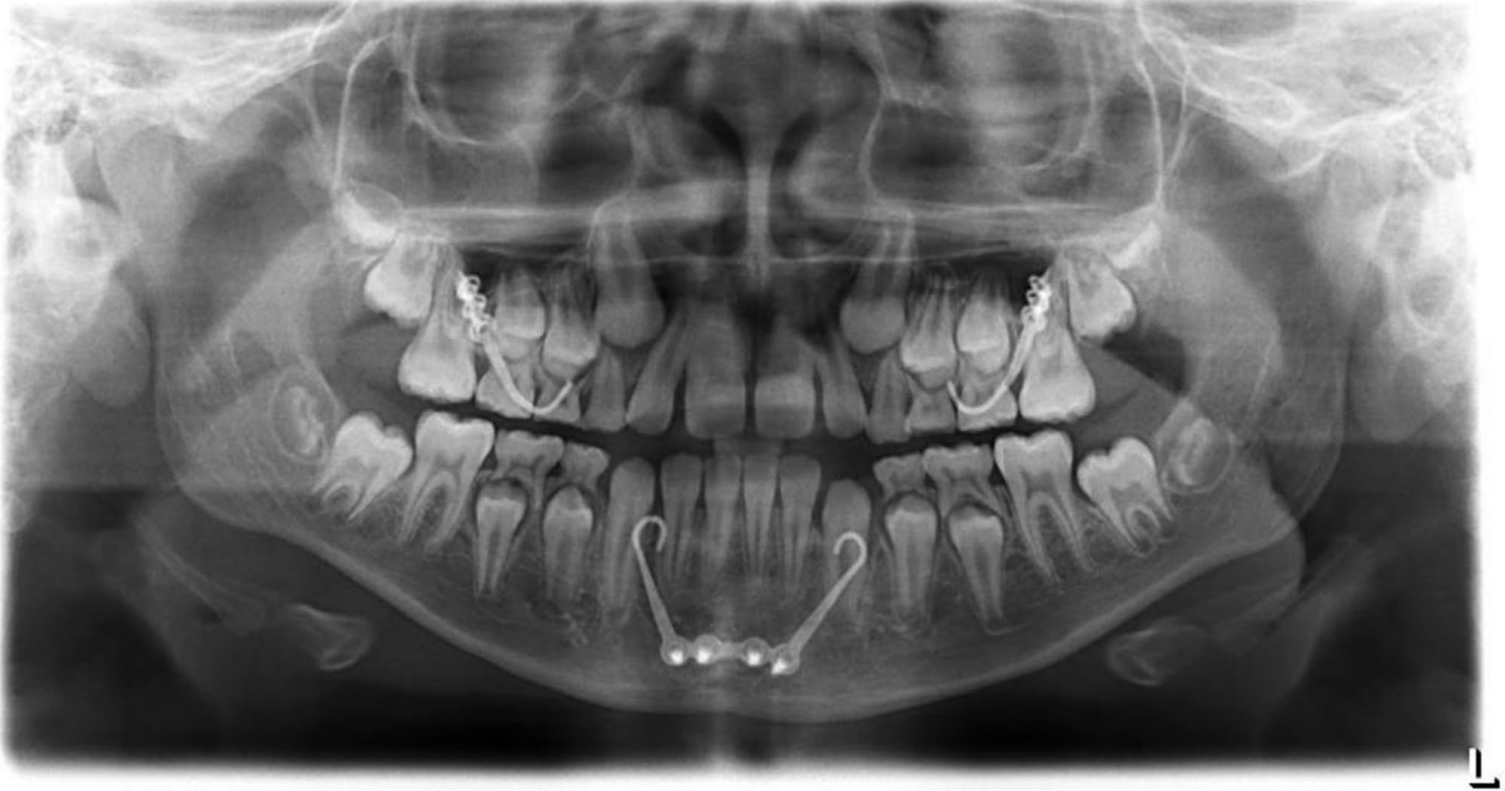
Panoramic radiograph with inserted bone anchors in upper and lower jaw on both sides

Four titanium miniplates (PSM Medical GmbH, Gunningen, Germany) were attached to the infrazygomatic crests and between the canine and lateral incisor of the mandibula on both sides under general anesthesia at the oral and maxillofacial surgery clinic at Saarland University Hospital (Figs. 1 and 2). The surgery was performed by two surgeons specialized in orthognathic surgery. Three weeks after the surgery, maxillomandibular class III elastics were engaged between the upper and lower bone anchor on each side. The initial force was 100 g per side. The elastics had to be changed by the patients at least once a day and they had to wear those 22 h per day. A removable bite plate was placed in the lower jaw to eliminate occlusal interference. The force of the elastics was increased to 250 g per side after two months. Transverse expansion of the maxilla was not performed, since all patients presented congruent dental arches including the transverse dimension. Active treatment time was 13.50 ± 5.87 months. After that, the patients were asked to wear the elastics only during the night for retention purposes for another six months.

### Treatment protocol of patients with conventional facemask

**Fig. 3:**
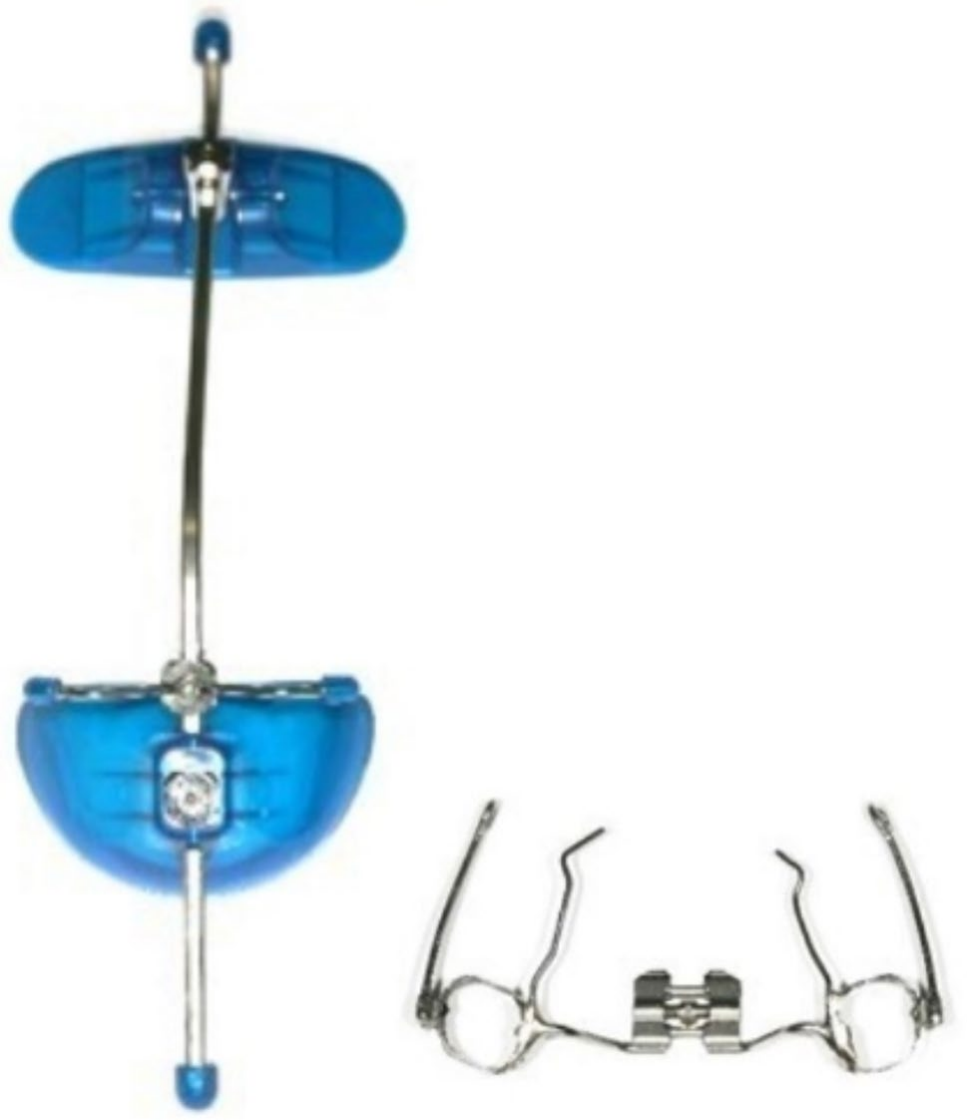
Conventional facemask and hyrax expander with hooks

A hyrax expander was inserted at the beginning of the treatment and if necessary, rapid maxillary expansion was performed by activation of the screw twice a day until the needed transverse dimension was achieved. Elastics were attached from hooks of the hyrax expander to the crossbar of the facemask (Fig. 3) and direction of elastic traction was forward and downward without interference of the lip. The force of the elastics was 350 g per side at the beginning and 500 g per side after two months. Patients were asked to wear the facemask 16 h per day. Removable bite plates were not needed. Active treatment time was 9.95 ± 4.17 months. After that, the patients were asked to wear the elastics only during the night for retention purposes for the remaining early orthodontic treatment time.

### Cephalometric measurement

A total of 62 cephalometric radiographs of patients with maxillary retrognathia and/or mandibular prognathia from one orthodontic clinic were available. A subdivision by gender was not performed. The cephalometric radiographs were measured by a single examiner using the software OnyxCeph^®^ 3TM (Image Instruments GmbH, Chemnitz, Germany).

### Landmarks and measuring technique

**Fig. 4:**
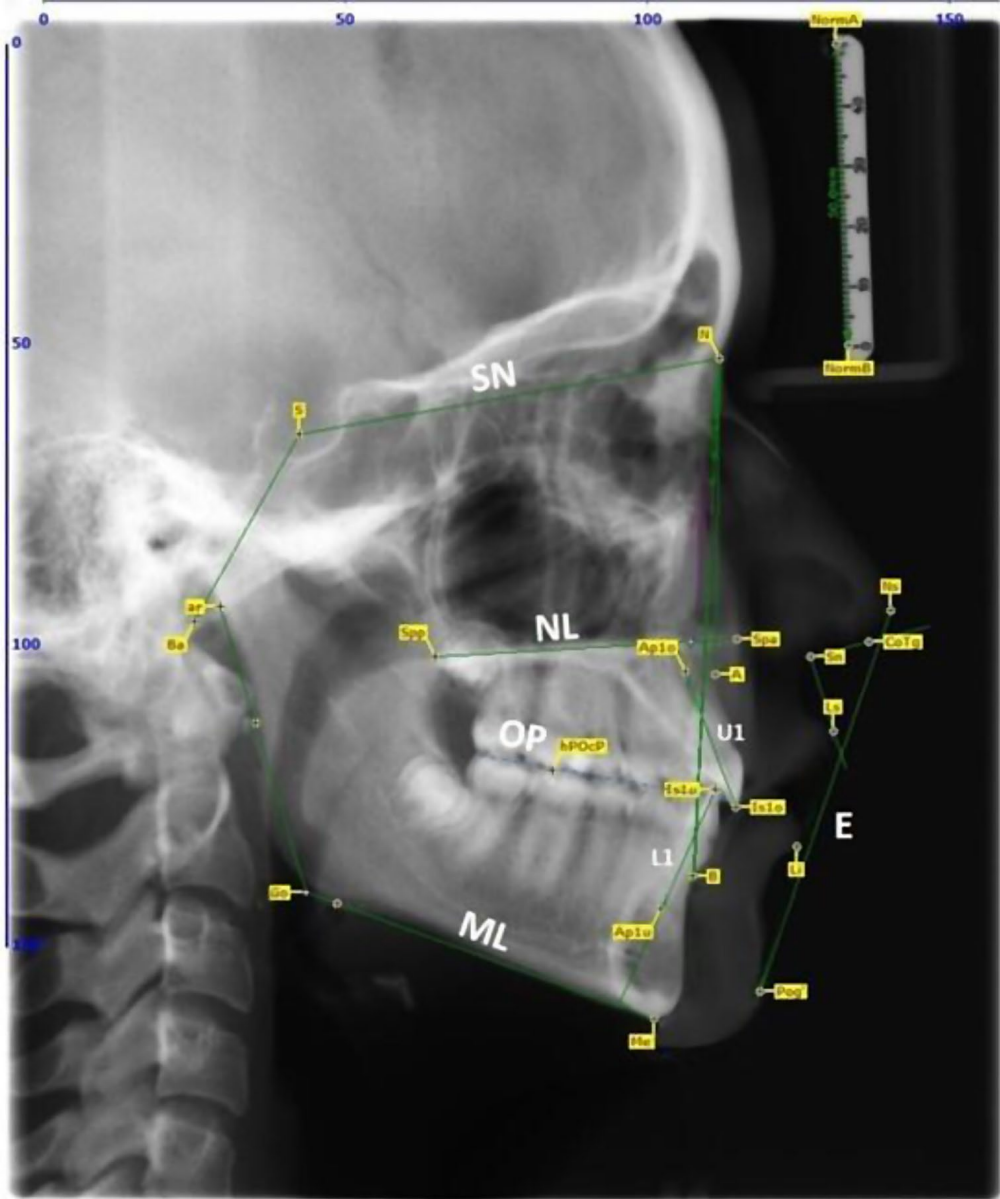
Overview of the landmarks used on the cephalometric radiographs and the linear and angular parameters calculated from them according to Schwarz and Segner and Hasund

The parameters for evaluation of the cephalometric radiographs were based on landmarks defined and used by and Schwarz [28] and Segner and Hasund [29] for calculating distances and angles (Table 1) in all groups (Fig. 4). The following landmarks were used for cephalometric analysis: Point NormA (NormA), NormB (Norm B), Nasion (N), Sella (S), Basion (Ba), Articulare (ar), Gonion (Go), Menton (Me), Point A (A), Point B (B), anterior nasal spine (Spa), posterior nasal spine (Spp), disto-buccal cuspid of the first lower molar (hPOcP), apical point of the upper incisor (Ap1o), incisal point of the upper incisor (Is1o), apical point of the lower incisor (Ap1u), incisal point of the lower incisor (Is1u), most anterior point of the soft tissue of the nose (Ns) with its septum (CoTg), Subnasale (Sn), most anterior point of the upper lip (Ls), most anterior point of the lower lip (Li) and most anterior point of the soft tissue of the chin (Pog´).

The angles SNA, SNB, ANB, NL-SN, ML-SN, ML-NL, MeGoAr and Wits appraisal were used to evaluate the sagittal and vertical position of maxilla and mandibula and the growth pattern. The angles U1-NL and L1-ML were used to evaluate the inclination of upper and lower incisors. The nasolabial angle and the distances ULE and LLE were used to evaluate the soft tissue of the nose, upper and lower lip. The angle OP-ML was used to evaluate the inclination of the occlusal plane.

**Table 1:** Cephalometric measurements and landmarks

*Measurements*	
**Distances (mm)**	
Wits	distance between the deepest point on the curvature of the anterior surface of the maxilla (Point A, (A)) and the deepest point on the curvature of the anterior surface of the mandibula (Point B, (B)) at occlusal plane level (distance between Point hPOcP and half of the distance of Point Is1o and Point Is1u)
ULE	distance between the most anterior point of the upper lip (Point Ls, (Ls)) and the distance (esthetic line, (E)) between the most anterior point of the soft tissue of the chin (Point Pog’, (Pog’)) and the most anterior point of the soft tissue of the nose (Point Ns, (Ns))
LLE	distance between the most anterior point of the lower lip (LL, (Li)) and the distance (esthetic line, (E)) between the most anterior point of the soft tissue of the chin (Point Pog’, (Pog’)) and the most anterior point of the soft tissue of the nose (Point Ns, (Ns))
**Angles (°)**	
SNA	angle between the cranial base (SN) and the deepest point on the curvature of the anterior surface of the maxilla (Point A, (A))
SNB	angle between the cranial base (SN) and the deepest point on the curvature of the anterior surface of the mandibula (Point B, (B))
ANB	angle between the deepest point on the curvature of the anterior surface of the maxilla (Point A, (A)), the deepest point of the nasofrontal suture (Nasion, (N)) and the deepest point on the curvature of the anterior surface of the mandibula (Point B, (B))
NL-SN	angle between the distance Spa-Spp (nasal line, (NL)) and the cranial base (SN)
ML-SN	angle between the mandibular plane (ML) and the cranial base (SN)
ML-NL	angle between the mandibular plane (ML) and the distance Spa-Spp (nasal line, (NL))
MeGoAr	gonial angle: angle between the most inferior point of the mandibular symphysis (Menton, (Me)), the most inferior posterior point of the mandibular angle (Gonion, (Go)) and the intersection of the dorsal contour of the condylar head and the contour of the posterior cranial base (Articulare, (Ar))
U1-NL	angle between the distance Ap1o-Is1o (longitudinal axis of the upper central incisor, (U1)) and the distance Spa-Spp (nasal line, (NL))
L1-ML	angle between the distance Ap1u-Is1u (longitudinal axis of the lower central incisor, (L1)) and the mandibular plane (ML)
Nasolabial	angle between the distance Ls-Sn and the distance Sn-CoTg
OP-ML	angle between the occlusal plane (OP) and the mandibular plane (ML)

### Statistical method, error of the method

Statistical analysis was performed with the SPSS software version 28 (IBM, Armonk, NY, USA). Statistics included Shapiro-Wilk- and T-Tests for the cephalometric radiographs. Paired samples T-Test was used for intragroup differences. Independent samples T-Test was used for intergroup differences. The level of significance was set at *p* < 0.05. The significance level was defined as follows: *p* ≥ 0.05 not significant, *p* < 0.05 significant, *p* < 0.01 highly significant and *p* < 0.001 most highly significant. The effect size was tested using Cohen´s criteria (for d): 0.2 = small effect size and low correlation, 0.5 = moderate effect size and correlation, 0.8 = large effect size and high correlation. For testing the interrater-reliability the evaluation process was repeated on 25% of each group two months after the first investigation to evaluate the impact of landmarking errors, which involved removing and replacing the markings. The differences were statistically analyzed using Dahlberg´s error of the method (MF) with the formula MF=√(∑d^2^/2n), where *d* is the difference between two measurement results and *n* is the number of duplicate measurements [9]. The MF for angular and linear measurements in the present study was < 1 for all measurements. Intrarater-reliability was not tested, since a single examiner conducted the investigation and the degree of subjectivity existing despite time-shift should be minimized.

## Results

### Cephalometric measurements

#### Bone anchor (Table 2)

**Table 2:** Bone anchors (*n* = 12) – cephalometric measurements: angles [°] and distances [mm]. t_0_: pretreatment visit; t_1_: posttreatment visit, *M* Mean, *SD* standard deviation, ^a^Paired samples T-test within group between t_0_-t_1_

*Angles*				
T	t0	t_1_	Δ	
	M ± SD	M ± SD	M ± SD	*P* value^a^
SNA	80.13 ± 3.93	82.43 ± 3.93	2.30 ± 1.18	< 0.001
SNB	81.63 ± 4.05	82.34 ± 4.38	0.71 ± 1.28	0.081
ANB	− 1.51 ± 1.40	0.08 ± 1.56	1.58 ± 1.32	0.002
NL-SN	6.58 ± 2.97	7.48 ± 2.37	0.90 ± 2.24	0.192
ML-SN	32.02 ± 4.26	31.01 ± 5.67	–1.01 ± 2.84	0.244
ML-NL	25.46 ± 3.13	23.36 ± 4.83	–2.10 ± 3.73	0.077
MeGoAr	125.08 ± 4.46	125.49 ± 6.36	0.41 ± 4.00	0.73
U1-NL	112.12 ± 6.91	113.81 ± 8.98	1.69 ± 5.62	0.32
L1-ML	85.29 ± 6.85	87.89 ± 7.95	2.03 ± 4.81	0.171
Nasolabial	99.25 ± 15.24	104.17 ± 10.18	4.92 ± 17.59	0.354
OP-ML	16.00 ± 4.00	17.00 ± 4.02	1.00 ± 3.05	0.14

*Distances*				
T	t_0_	t_1_	Δ	
	M ± SD	M ± SD	M ± SD	P value^a^
Wits	− 5.43 ± 1.70	–3.43 ± 2.31	2.01 ± 2.65	0.032
ULE	− 4.25 ± 2.67	–4.83 ± 2.95	− 0.58 ± 2.07	0.349
LLE	− 1.75 ± 2.34	–3.17 ± 2.98	–1.42 ± 2.02	0.033

##### Angles (Fig. 5)

**Fig. 5:**
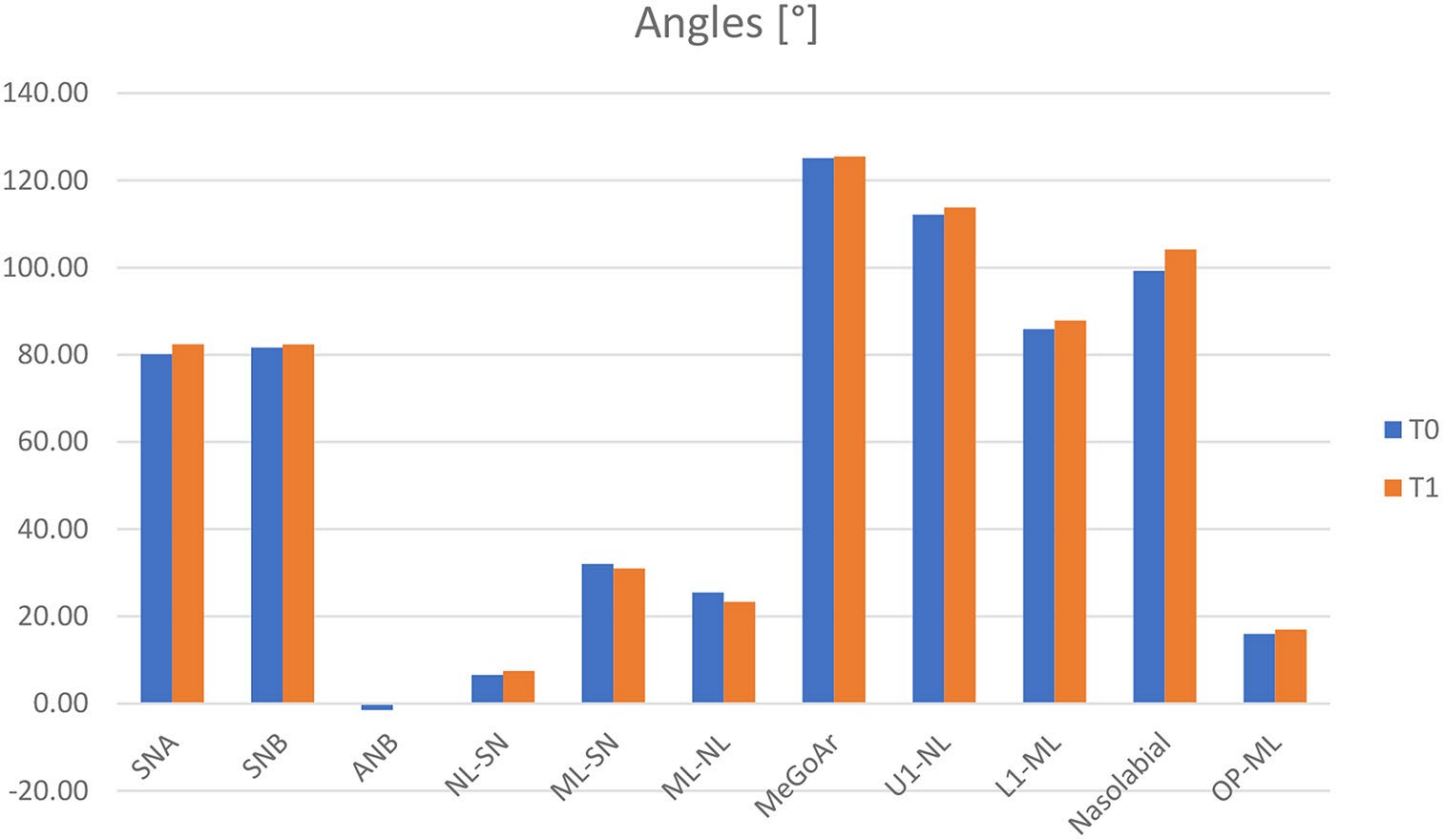
Bone anchors (*n* = 12) – cephalometric measurements: angles [°]. t_0_: pretreatment visit; t_1_: posttreatment visit

The changes between t_0_ and t_1_ showed active treatment effects. SNA angle showed a significant increase of 2.30 ± 1.18° (t_0_: 80.13 ± 3.93°; t_1_: 82.43 ± 3.93°; p = < 0.001; d = 1.179). The maxilla moved forward. SNB angle showed an increase of 0.71 ± 1.28° (t_0_: 81.63 ± 4.05°; t_1_: 82.34 ± 4.38°; *p* = 0.081). The mandibula moved forward. According to the changes of the maxilla and mandibula, ANB angle showed a significant increase of 1.58 ± 1.32° (t_0_: -1.51 ± 1.40°; t_1_: 0.08 ± 1.56°; *p* = 0.002; d = 1.324). NL-SN angle showed an increase of 0.90 ± 2.24° (t_0_: 6.58 ± 2.97°; t_1_: 7.48 ± 2.37°; *p* = 0.192). The maxilla rotated clockwise. ML-SN angle showed a decrease of -1.01 ± 2.84° (t_0_: 32.02 ± 4.26°; t_1_: 31.01 ± 5.67°; *p* = 0.244). The mandibula rotated counterclockwise. ML-NL angle showed a decrease of -2.10 ± 3.73° (t_0_: 25.46 ± 3.13°; t_1_: 23.36 ± 4.83°; *p* = 0.077) because of the rotation of the maxilla and mandibula. MeGoAr angle showed an increase of 0.41 ± 4.00° (t_0_: 125.08 ± 4.46°; t_1_: 125.49 ± 6.36°; *p* = 0.730). The difference was almost indistinguishable. U1-NL angle showed an increase of 1.69 ± 5.62° (t_0_: 112.12 ± 6.91°; t_1_: 113.81 ± 8.98°; *p* = 0.320). A proclination was recorded for the maxillary incisors. L1-ML angle showed an increase of 2.03 ± 4.81° (t_0_: 85.29 ± 6.85°; t_1_: 87.89 ± 7.95°; *p* = 0.171). A proclination was recorded for the mandibular incisors as well. Nasolabial angle showed an increase of 4.92 ± 17.59° (t_0_: 99.25 ± 15.24°; t_1_: 104.17 ± 10.18°; *p* = 0.354). The upper lip moved forward due to maxillary and upper incisor changes. OP-ML angle showed an increase of 1.00 ± 3.05° (t_0_: 16.00 ± 4.00°; t_1_: 17.00 ± 4.02°; *p* = 0.140).

##### Distances (Fig. 6)

**Fig. 6:**
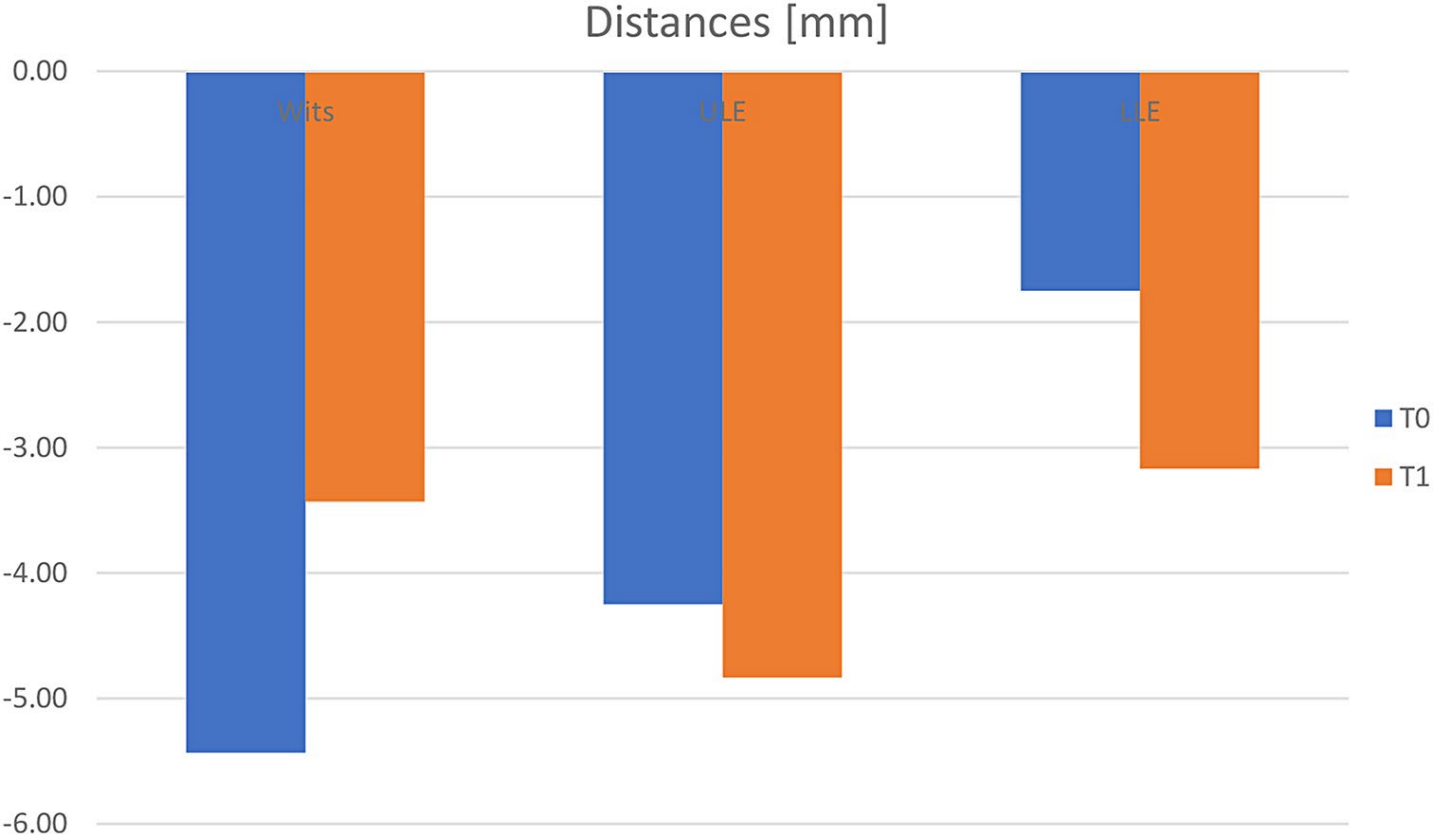
Bone anchors (*n* = 12) – cephalometric measurements: distances [mm]. t_0_: pretreatment visit; t_1_: posttreatment visit

Wits appraisal showed a significant increase of 2.01 ± 2.65 mm (t_0_: -5.43 ± 1.70 mm; t_1_: -3.43 ± 2.31 mm; *p* = 0.032; d = 2.442) because of skeletal and dental changes of the maxilla and the mandibula. The distance ULE showed a decrease of -0.58 ± 2.07 mm (t_0_: -4.25 ± 2.67 mm; t_1_: -4.83 ± 2.95 mm; *p* = 0.349). The difference was almost indistinguishable.

The distance LLE showed a significant decrease of -1.42 ± 2.02 mm (t_0_: -1.75 ± 2.34 mm; t_1_: -3.17 ± 2.98; *p* = 0.033; d = 2.021). The lower lip moved backward.

#### Facemask (Table 3)

**Table 3:** Conventional facemask (*n* = 19) – cephalometric measurements: angles [°] and distances [mm]. t_0_: pretreatment visit; t_1_: posttreatment visit, *M* Mean, *SD* standard deviation, ^a^Paired samples T-test within group between t_0_-t_1_

*Angles*				
T	t_0_	t_1_	Δ	
	M ± SD	M ± SD	M ± SD	*P* value^a^
SNA	80.30 ± 3.40	81.52 ± 3.49	1.22 ± 2.28	0.031
SNB	80.12 ± 2.58	79.31 ± 2.56	–0.81 ± 1.35	0.017
ANB	0.16 ± 2.20	2.20 ± 2.56	2.04 ± 1.55	< 0.001
NL-SN	6.88 ± 3.07	6.25 ± 3.16	–0.64 ± 1.88	0.156
ML-SN	32.93 ± 4.04	33.98 ± 4.02	1.05 ± 1.51	0.007
ML-NL	26.06 ± 4.04	27.74 ± 3.97	1.68 ± 1.77	< 0.001
MeGoAr	128.13 ± 5.77	128.53 ± 6.61	0.40 ± 2.80	0.542
U1-NL	104.01 ± 8.33	107.35 ± 4.77	3.35 ± 6.18	0.03
L1-ML	88.06 ± 8.27	83.33 ± 7.47	–4.73 ± 5.28	0.001
Nasolabial	108.63 ± 10.09	106.05 ± 10.97	–2.58 ± 9.47	0.251
OP-ML	14.11 ± 4.03	16.74 ± 4.17	2.63 ± 2.43	< 0.001

*Distances*				
T	t_0_	t_1_	Δ	
	M ± SD	M ± SD	M ± SD	*P* value^a^
Wits	−3.83 ± 3.22	–1.98 ± 2.23	1.85 ± 4.09	< 0.001
ULE	−2.26 ± 2.54	–1.32 ± 2.00	0.95 ± 1.90	0.043
LLE	0.05 ± 2.57	0.47 ± 2.63	0.42 ± 1.39	0.202

##### Angles (Fig. 7)

**Fig. 7:**
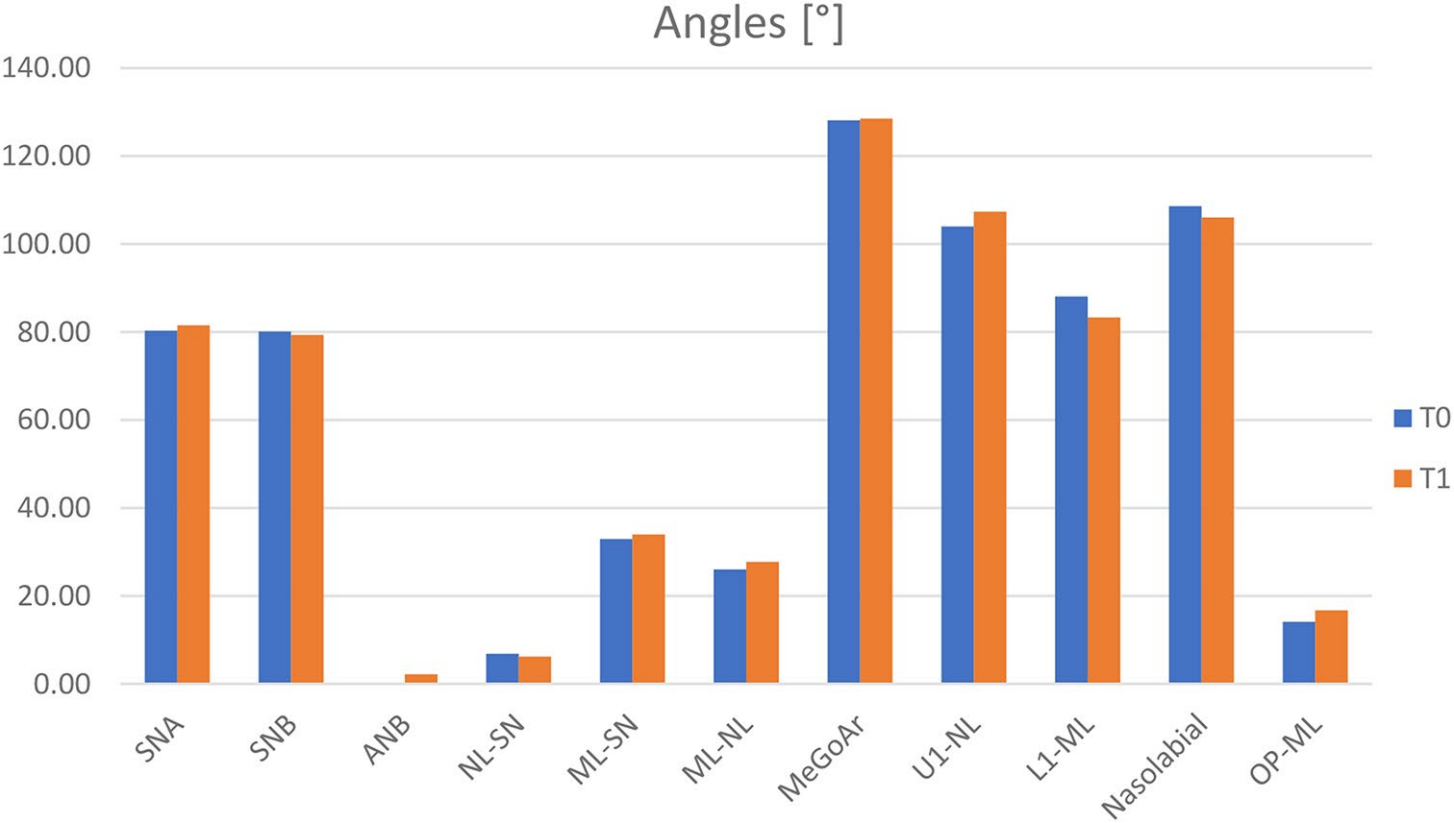
Conventional facemask (*n* = 19) – cephalometric measurements: angles [°]. t_0_: pretreatment visit; t_1_: posttreatment visit

In this group the changes between t_0_ and t_1_ showed active treatment effects, too. SNA angle showed a significant increase of 1.22 ± 2.28° (t_0_: 80.30 ± 3.40°; t_1_: 81.52 ± 3.49°; *p* = 0.031; d = 2.280). The maxilla moved forward. SNB angle showed a significant decrease of -0.81 ± 1.35° (t_0_: 80.12 ± 2.58°; t_1_: 79.31 ± 2.56°; *p* = 0.017; d = 1.348). The mandibula moved backward. According to the changes of the maxilla and mandibula, ANB angle showed a significant increase of 2.04 ± 1.55° (t_0_: 0.16 ± 2.20°; t_1_: 2.20 ± 2.56°; p = < 0.001; d = 1.547). NL-SN angle showed a decrease of -0.64 ± 1.88° (t_0_: 6.88 ± 3.07°; t_1_: 6.25 ± 3.16°; *p* = 0.156). The maxilla rotated counterclockwise. ML-SN angle showed a significant increase of 1.05 ± 1.51° (t_0_: 32.93 ± 4.04°; t_1_: 33.98 ± 4.02°; *p* = 0.007; d = 1.514). The mandibula rotated clockwise. ML-NL angle showed a significant increase of 1.68 ± 1.77° (t_0_: 26.06 ± 4.04°; t_1_: 27.74 ± 3.97°; p = < 0.001; d = 1.766) as a result of the rotation of the maxilla and mandibula. MeGoAr angle showed an increase of 0.40 ± 2.80° (t_0_: 128.13 ± 5.77°; t_1_: 128.53 ± 6.61°; *p* = 0.542). The difference was almost indistinguishable. U1-NL angle showed a significant increase of 3.35 ± 6.18° (t_0_: 104.01 ± 8.33°; t_1_: 107.35 ± 4.77°; *p* = 0.030; d = 6.184). A proclination was recorded for the maxillary incisors. L1-ML angle showed a significant decrease of -4.73 ± 5.28° (t_0_: 88.06 ± 8.27°; t_1_: 83.33 ± 7.47°; *p* = 0.001; d = 5.281). A retroclination was recorded for the mandibular incisors. Nasolabial angle showed a decrease of -2.58 ± 9.47° (t_0_: 108.63 ± 10.09°; t_1_: 106.05 ± 10.97°; *p* = 0.251). The upper lip moved backward. OP-ML angle showed a significant increase of 2.63 ± 2.43° (t_0_: 14.11 ± 4.03°; t_1_: 16.74 ± 4.17°; p = < 0.001; d = 2.432).

##### Distances (Fig. 8)

**Fig. 8:**
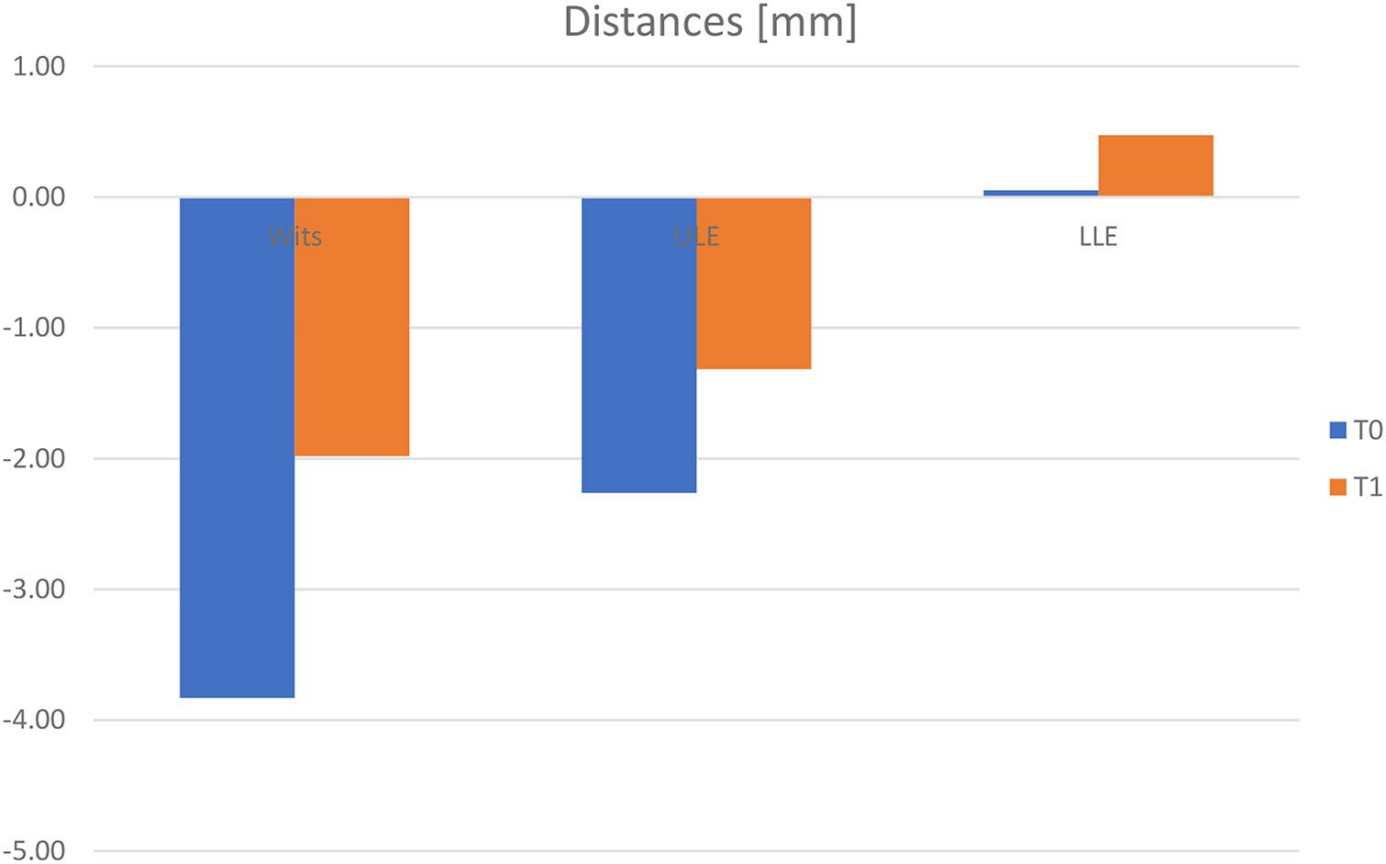
Conventional facemask (*n* = 19) – cephalometric measurements: distances [mm]. t_0_: pretreatment visit; t_1_: posttreatment visit

Wits appraisal showed a significant increase of 1.85 ± 4.09 mm (t_0_: -3.83 ± 3.22 mm; t_1_: -1.98 ± 2.23 mm; p = < 0.001; d = 1.705) because of skeletal and dental changes of the maxilla and the mandibula. The distance ULE showed a significant increase of 0.95 ± 1.90 mm (t_0_: -2.26 ± 2.54 mm; t_1_: -1.32 ± 2.00 mm; *p* = 0.043; d = 1.900). The difference was almost indistinguishable.

The distance LLE showed an increase of 0.42 ± 1.39 mm (t_0_: 0.05 ± 2.57 mm; t_1_: 0.47 ± 2.63; *p* = 0.202). The difference was almost indistinguishable, too.

#### Bone anchors versus conventional facemask – pretreatment results (Table 4)

**Table 4:** Bone anchors (*n* = 12) versus conventional facemask (*n* = 19) – cephalometric measurements: angles [°] and distances [mm]. t_0_: pretreatment visit, *M* Mean, *SD* standard deviation, ^a^Independent samples T-test between groups at t_0_

*Angles*				
	Bone anchors	Facemask		
	M ± SD	M ± SD	Δ	*P* value^a^
SNA	80.13 ± 3.93	80.30 ± 3.40	−0.17	0.896
SNB	81.63 ± 4.05	80.12 ± 2.58	+ 1.51	0.212
ANB	−1.51 ± 1.40	0.16 ± 2.20	–1.67	0.026
NL-SN	6.58 ± 2.97	6.88 ± 3.07	–0.30	0.79
ML-SN	32.02 ± 4.26	32.93 ± 4.04	–0.91	0.552
ML-NL	25.46 ± 3.13	26.06 ± 4.04	–0.60	0.665
MeGoAr	125.08 ± 4.46	128.13 ± 5.77	–3.05	0.131
U1-NL	112.12 ± 6.91	104.01 ± 8.33	+ 8.11	0.009
L1-ML	85.29 ± 6.85	88.06 ± 8.27	–2.77	0.449
Nasolabial	99.25 ± 15.24	108.63 ± 10.09	–9.38	0.048
OP-ML	16.00 ± 4.00	14.11 ± 4.03	+ 1.89	0.105

*Distances*				
	Bone anchors	Facemask		
	M ± SD	M ± SD	Δ	*P* value^a^
Wits	−5.43 ± 1.70	–3.83 ± 3.22	–1.60	0.227
ULE	−4.25 ± 2.67	–2.26 ± 2.54	–1.99	0.046
LLE	−1.75 ± 2.34	0.05 ± 2.57	–1.80	0.059

##### Angles

SNA angle was smaller in patients with bone anchors (BAP) than in patients with facemask (FP) (BAP: 80.13 ± 3.93°; FP: 80.30 ± 3.40°; Δ: -0.17; *p* = 0.896). SNB angle was larger in patients with bone anchors (BAP) than in patients with facemask (FP) (BAP: 81.63 ± 4.05°; FP: 80.12 ± 2.58°; Δ: +1.51; *p* = 0.212). ANB angle was significantly smaller in patients with bone anchors (BAP) than in patients with facemask (FP) (BAP: -1.51 ± 1.40°; FP: 0.16 ± 2.20°; Δ: -1.67; *p* = 0.026; d = 1.936). NL-SN angle was smaller in patients with bone anchors (BAP) than in patients with facemask (FP) (BAP: 6.58 ± 2.97°; FP: 6.88 ± 3.07°; Δ: -0.30; *p* = 0.790). ML-SN angle was smaller in patients with bone anchors (BAP) than in patients with facemask (FP) (BAP: 32.02 ± 4.26°; FP: 32.93 ± 4.04°; Δ: -0.91; *p* = 0.552). ML-NL angle was smaller in patients with bone anchors (BAP) than in patients with facemask (FP) (BAP: 25.46 ± 3.13°; FP: 26.06 ± 4.04°; Δ: -0.60; *p* = 0.665. MeGoAr angle was smaller in patients with bone anchors (BAP) than in patients with facemask (FP) (BAP: 125.08 ± 4.46°; FP: 128.13 ± 5.77°; Δ: -3.05; *p* = 0.131). U1-NL angle was significantly larger in patients with bone anchors (BAP) than in patients with facemask (FP) (BAP: 112.12 ± 6.91°; FP: 104.01 ± 8.33°; Δ: +8.11; *p* = 0.009; d = 7.761). L1-ML angle was smaller in patients with bone anchors (BAP) than in patients with facemask (FP) (BAP: 85.29 ± 6.85°; FP: 88.06 ± 8.27°; Δ: -2.77; *p* = 0.449). Nasolabial angle was significantly smaller in patients with bone anchors (BAP) than in patients with facemask (FP) (BAP: 99.25 ± 15.24°; FP: 108.63 ± 10.09°; Δ: -9.38; *p* = 0.048; d = 12.299).

OP-ML angle was larger in patients with bone anchors (BAP) than in patients with facemask (FP) (BAP: 16.00 ± 4.00°; FP: 14.11 ± 4.03°; Δ: +1.89; *p* = 0.105).

##### Distances

Wits was smaller in patients with bone anchors (BAP) than in patients with facemask (FP) (BAP: -5.43 ± 1.70 mm; FP: -3.83 ± 3.22 mm; Δ: -1.60; *p* = 0.227).

The distance ULE was significantly smaller in patients with bone anchors (BAP) than in patients with facemask (FP) (BAP: -4.25 ± 2.67; FP: -2.26 ± 2.54 mm; Δ: -1.99; *p* = 0.046; d = 2.486). The distance LLE was smaller in patients with bone anchors (BAP) than in patients with facemask (FP) (BAP: -1.75 ± 2.34; FP: 0.05 ± 2.57 mm; Δ: -1.80; *p* = 0.059).

#### Bone anchors versus conventional facemask – posttreatment results (Table 5)

**Table 5:** Bone anchors (*n* = 12) versus conventional facemask (*n* = 19) – cephalometric measurements: angles [°] and distances [mm]. t_1_: posttreatment visit, *M* Mean, *SD* standard deviation, ^a^Independent samples T-test between groups at t_1_

*Angles*				
	Bone anchors	Facemask		
	M ± SD	M ± SD	Δ	P value^a^
SNA	82.43 ± 3.93	81.52 ± 3.49	+ 0.91	0.508
SNB	82.34 ± 4.38	79.31 ± 2.56	+ 3.03	0.021
ANB	0.08 ± 1.56	2.20 ± 2.56	–2.12	0.015
NL-SN	7.48 ± 2.37	6.25 ± 3.16	+ 1.23	0.255
ML-SN	31.01 ± 5.67	33.98 ± 4.02	–2.98	0.049
ML-NL	23.36 ± 4.83	27.74 ± 3.97	–4.38	0.01
MeGoAr	125.49 ± 6.36	128.53 ± 6.61	–3.04	0.217
U1-NL	113.81 ± 8.98	107.35 ± 4.77	+ 6.46	0.014
L1-ML	87.89 ± 7.95	83.33 ± 7.47	+ 4.56	0.117
Nasolabial	104.17 ± 10.18	106.05 ± 10.97	–1.88	0.635
OP-ML	17.00 ± 4.02	16.74 ± 4.17	+ 0.26	0.432

Distances				
	Bone anchors	Facemask		
	M ± SD	M ± SD	Δ	P value^a^
Wits	− 3.43 ± 2.31	–1.98 ± 2.23	–1.45	0.06
ULE	− 4.83 ± 2.95	–1.32 ± 2.00	-3.51	< 0.001
LLE	− 3.17 ± 2.98	0.47 ± 2.63	–3.64	0.001

##### Angles

In both groups the changes at t_1_ showed active treatment results. SNA angle was larger in patients with bone anchors (BAP) than in patients with facemask (FP) (BAP: 82.43 ± 3.93°; FP: 81.52 ± 3.49°; Δ: +0.91; *p* = 0.508). SNB angle was significantly larger in patients with bone anchors (BAP) than in patients with facemask (FP) (BAP: 82.34 ± 4.38°; FP: 79.31 ± 2.56°; Δ: +3.03; *p* = 0.021; d = 3.372). ANB angle was significantly smaller in patients with bone anchors (BAP) than in patients with facemask (FP) (BAP: 0.08 ± 1.56°; FP: 2.20 ± 2.56°; Δ: -2.12; *p* = 0.015; d = 2.237). NL-SN angle was larger in patients with bone anchors (BAP) than in patients with facemask (FP) (BAP: 7.48 ± 2.37°; FP: 6.25 ± 3.16°; Δ: +1.23; *p* = 0.255). ML-SN angle was significantly smaller in patients with bone anchors (BAP) than in patients with facemask (FP) (BAP: 31.01 ± 5.67°; FP: 33.98 ± 4.02°; Δ: -2.98; *p* = 0.049; d = 4.714). ML-NL angle was significantly smaller in patients with bone anchors (BAP) than in patients with facemask (FP) (BAP: 23.36 ± 4.83°; FP: 27.74 ± 3.97°; Δ: -4.38; *p* = 0.010; d = 4.314). MeGoAr angle was smaller in patients with bone anchors (BAP) than in patients with facemask (FP) (BAP: 125.49 ± 6.36°; FP: 128.53 ± 6.61°; Δ: -3.04; *p* = 0.217). U1-NL angle was significantly larger in patients with bone anchors (BAP) than in patients with facemask (FP) (BAP: 113.81 ± 8.98°; FP: 107.35 ± 4.77°; Δ: +6.46; *p* = 0.014; d = 6.685). L1-ML angle was larger in patients with bone anchors (BAP) than in patients with facemask (FP) (BAP: 87.89 ± 7.95°; FP: 83.33 ± 7.47°; Δ: +4.56; *p* = 0.117). Nasolabial angle was smaller in patients with bone anchors (BAP) than in patients with facemask (FP) (BAP: 104.17 ± 10.18°; FP: 106.05 ± 10.97°; Δ: -1.88; *p* = 0.635). OP-ML angle was larger in patients with bone anchors (BAP) than in patients with facemask (FP) (BAP: 17.00 ± 4.02°; FP: 16.74 ± 4.17°; Δ: +0.26; *p* = 0.432).

##### Distances

Wits was smaller in patients with bone anchors (BAP) than in patients with facemask (FP) (BAP: -3.43 ± 2.31 mm; FP: -1.98 ± 2.23 mm; Δ: -1.45; *p* = 0.060).

The distance ULE was significantly smaller in patients with bone anchors (BAP) than in patients with facemask (FP) (BAP: -4.83 ± 2.95; FP: -1.32 ± 2.00 mm; Δ: -3.51; p = < 0.001; d = 2.405). The distance LLE was significantly smaller in patients with bone anchors (BAP) than in patients with facemask (FP) (BAP: -3.17 ± 2.98; FP: 0.47 ± 2.63 mm; Δ: -3.64; *p* = 0.001; d = 2.769).

#### Bone anchors complications

Local infection with granulation of the mucosa was seen in five patients in the upper jaw on both sides and in one patient in the lower jaw on both sides. In eight patients all miniplates remained stable throughout protraction. Loosening rate was 14.58% with two patients on both sides, one patient on the left side and two patients on the right side of the upper jaw. In four patients with loosening of the bone anchors, the bone anchors were removed and replaced three months after removal. In one patient with loosening of the bone anchor on the left side, loosening was low and elastic wear was still possible after consultation with the surgeons. Loosening was recognized in four patients after three months of elastic wear and in one patient after four months of elastic wear.

## Discussion

Bhatia and Leighton [3] described natural growth of the maxilla at the age of ten to twelve years by an increase of SNA angle of 0.5° and natural growth of the mandibula at that age by an increase of SNB angle of 0.6°. Modifying facial growth using orthopedic forces has been of special interest in orthodontists for ages, since maxillary protraction was pioneered by Delaire in the 1970s [14]. Forward movement of the maxilla by 1–2 mm, a 3° increase in SNA, 1.02° decrease in SNB and a reduction in ANB of -2.43° were described with wide variations [5, 8, 10, 14, 18]. Improved dental arch relationships mostly because of dentoalveolar compensation were the results of maxillary protraction with facemask [11]. In literature, the ideal treatment timing for orthopedic treatment of class III malocclusion with maxillary retrognathia was at the age of five to eight years during deciduous and early mixed dentition [14, 23]. Six months after maxillary protraction, the maxilla showed forward movement, but also proclination of upper incisors and extrusion of maxillary molars resulting in increased lower face height and the mandibula rotating in posterior direction. Correction of the class III malocclusion was due to forward movement of the maxilla, but also clockwise rotation of the mandibula. Therefore, correction of the overjet was because of dental and skeletal changes [19, 23]. Unwanted dental and skeletal side effects, such as proclination of upper incisors and clockwise rotation of the mandibula, were also seen in patients with facemask of our study. Success and failure of class III malocclusion treatment depends on the potential of growth and treatment is requiring a long-term period, making patient´s motivation difficult in the long run [23, 30]. Therefore, knowing the ideal timing for facemask therapy to obtain better treatment results is indispensable. Takada et al. [30] described the maximum peak of maxillary growth between ten to twelve years of age for girls and twelve to thirteen years for boys. This claim is contentious. Other than that, Alexander et al. suggested that the maximum peak of maxillary growth occurs during the prepubertal period [1]. Orthopedic class III malocclusion treatment shows the best results when the facemask is applied before the pubertal growth spurt, because the suture´s adaptability and response to maxillary protraction decreases with age [11, 24, 30]. Dibbets and van der Weele [15] investigated the treatment with the facemask and its forces regarding temporomandibular joint dysfunction. They reported no causal relationship of facemask treatment with temporomandibular joint dysfunction even with a 500 g force on each side. In our study, neither patients with bone anchors nor patients with facemask showed symptoms of temporomandibular joint disfunction before or after treatment. To reduce unwanted side effects of facemask therapy, titanium bone anchors were used some years later for maxillary protraction being well tolerated by the patients [6, 10, 11]. Maxillary protraction was performed using a rigid external distractor, a facemask after Le Fort I corticotomy in patients with a cleft or a combination of skeletal anchorage in the upper jaw and facemask [10]. Liu et al. [22] described a technique using bone anchored hooks combined with facemask and additional sutural distraction for correction in four patients at the age of six to twelve years or Le Fort III osteotomy in four patients older than twelve years with and without cleft lip and palate. No complications concerning surgery process or loosening of the bone-born hooks for distraction occurred. The midface advancement was 8 mm in patients with sutural distraction and 10 mm in patients with Le Fort III osteotomy with remarkable changes in face contour and normal occlusion after the treatment. There was no relapse described after a six months follow-up. Kircelli and Pektas [21] used miniplates on the lateral nasal wall of the maxilla in six patients at the mean age of 11.8 ± 1.1 years and attached them to a facemask for 10.8 ± 2.4 months. The infraorbital region moved 3.3 ± 1.1 mm forward. Point A moved 4.8 ± 2.0 mm forward. The results remained stable over the 15.2 ± 0.9 months follow-up period. They concluded that skeletal anchorage combined with a facemask leads to remarkable advancement of the midface and soft-tissue profile in the late mixed-dentition period. Bone anchors and class III elastics without additional corticotomy or osteotomy was pioneered by De Clerck et al. in the 2000s [11]. Extraoral facemask was no longer needed with this approach and elastics can be worn all over the day. In this study, De Clerck et al. described the treatment of three female patients with maxillary deficiency and a concave soft tissue profile at the age of ten to eleven years. Anterior crossbite was corrected in all three patients after treatment and the soft tissue profile improved. The cephalometric radiographs showed an improvement of ANB, Wits appraisal and facial convexity. Upper incisor inclination remained stable during treatment, lower incisors were proclined afterwards. The class III correction was stable from the end of treatment to a 11- and 38-months follow-up. In a later study, De Clerck et al. [13] treated twenty-five Class III patients at the mean age of 11.10 ± 1.1 years with bone anchors and Class III elastics and took cone-beam computed tomography images before elastic wear and after treatment. They reported a posterior displacement of the mandibula after the treatment in all patients. The displacement of the posterior ramus was 2.74 ± 1.36 mm, of the condyles, 2.07 ± 1.16 mm and of the chin − 0.13 ± 2.89 mm. Even remodeling of the mandibular fossa at its anterior eminence was 1.38 ± 1.03 mm and resorption of bone of the posterior region was − 1.34 ± 0.06 mm. Cevidanes et al. [4] compared 21 patients with bone anchors at the mean age of 11 years 10 months ± 1 year 10 months and 34 patients with facemask at the mean age of 8 years 3 months ± 1 year 10 months after one year of treatment. Maxillary advancement and midfacial length were about 2.5–3.0 mm larger in patients with bone-anchors. There were no differences between sagittal growth and position of the mandibula between the two groups. Maxillomandibular divergency was decreased of about 3° in patients with bone anchors, slight counterclockwise rotation of the mandibula was noted in patients with bone anchors and clockwise rotation of the mandibula was seen in patients with facemask. Patients with bone anchors did not show the same amount of lingual inclination of lower incisors as patients with facemask did. Nguyen et al. [26] reported their results of twenty-five Class III patients at the mean age of 11.10 ± 1.1 years treated with bone anchors and Class III elastics. Cone-beam computed tomography images before elastic wear and after treatment showed a mean forward displacement of the maxilla of 3.7 mm and of the zygomas of 4.3 mm, but also incisors came forward 3.7 mm. De Clerck and Swennen [10] described the success rate of miniplates concerning stability with 97% in twenty-five patients with mean age 12.0 ± 1.2 years, but during elastic use five miniplates out of hundred showed signs of mobility. Two miniplates were stable again, after the patients stopped using elastics for two months. The other three miniplates were removed and replaced after three months of healing. Contrary to expectations Cornelis et al. [7] concluded in their systematic review of 28 full-text articles concerning bone-anchored maxillary protraction that the level of evidence available for supporting maxillary protraction effect using bone anchors was low. They remarked identical samples in publications reporting results that tended to suggest positive results using bone anchors for class III malocclusion treatment. They even questioned clinical significance concerning the differences in sagittal correction between bone anchors and facemask and recommended long-term follow-up results. In a newer study Kamel et al. [20] reported their results of seventeen Class III patients during late mixed or early permanent dentition treated with a hybrid hyrax expander and class III elastics to a bone-supported bar in the mandibula for about one year compared to thirteen patients without treatment. SNA angle showed an increase in the treated group of 4.64 ± 0.95° and in the control group of 0.42 ± 0.21°. SNB angle showed a decrease in the treated group of -0.25 ± 0.47° and in the control group an increase of 1.03 ± 0.59°. ANB angle showed an increase in the treated group of 4.90 ± 1.31° and a decrease in the control group of -0.61 ± 0.55°. Wits appraisal showed an increase in the treated group of 5.27 ± 1.07° and in the control group of 0.28 ± 0.45°. The lower face height increased in the treated group and the mandibula showed a clockwise rotation with closure of gonial angle. Maxillary and mandibular incisors showed proclination in the treated group. Mesialization and extrusion of upper molars were seen in the treated group. Nienkemper et al. [27] reported their results of 16 growing class III children (mean age 9.5 ± 1.6 years) treated with a hybrid hyrax-facemask combination using pre- and posttreatment cephalograms compared with a control group of 16 untreated Class III subjects. The mean treatment duration was 5.8 ± 1.6 months. The results showed significant improvement in SNA (2.4°), SNB (-1.7°) and Wits appraisal (4.5 mm). Comparison of the treatment and the control group showed a larger gonial angle in the control group. All mini-implants in the treatment group remained stable during treatment. Compared to the results of our study, these values were higher than in our facemask group, since we did not use mini-implants. Ngan et al. [25] compared 20 class III patients (mean age 9.8 ± 1.6 years) with tooth-borne rapid palatal expansion appliance and facemask and 20 class III patients (mean age 9.6 ± 1.2 years) with bone-anchored rapid palatal expansion appliance and facemask. The tooth-borne facemask group showed more proclination of maxillary incisors (2.12 mm), the bone-anchored facemask group showed less downward movement of Point A (-0.4 mm) than the tooth-borne facemask group (1.2 mm) and less opening of the mandibular plane in the bone-anchored facemask group (-0.25°) than in the tooth-borne facemask group (2.76°). The results of the tooth-borne facemask group were comparable to the results of our study. Regarding failure rate, the hybrid-hyrax with palatal mini-screws could be an alternative to the bone anchors used in our study. However, treatment with mini-screws is not covered by the statutory health insurance (GKV) applying to our patients. Since most of those were dependent on treatment modalities covered by the statutory health insurance, they decided on bone anchors as described in our study.

The observed failure of the bone anchors was mainly due to poor oral or hand hygiene, which led to infections and subsequent loosening of the bone anchors. Therefrom, better hygiene could lower the failure rate.

Early treatment of class III patients, as shown in our study at the age of 6.74 ± 1.15 years for facemask patients, aimed to effect maxillary growth at sutures articulating with the frontal, zygomatic, ethmoid and palatal bones. Later treatment of class III patients, as shown in our study at the age of 11.00 ± 1.76 years for bone anchor patients, aimed to effect apposition processes over all surfaces, as this is the predominant growth mechanism after the end of suture activity at around seven years of age [16].

In both groups, changes at t1 showed active treatment outcomes of the facemask or bone anchors, including possible growth effects that may occur between six and eleven years of age. Nonetheless, the maxilla moved more forward in patients with bone anchors (2.30 ± 1.18°) than in patients with facemask (1.22 ± 2.28°). Contrary to expectations, the mandibula moved more forward in patients with bone anchors (0.71 ± 1.28°) than in patients with facemask (-0.81 ± 1.35°) as well. Accordingly, the ANB angle was smaller in patients with bone anchors (0.08 ± 1.56°) than in patients with facemask (2.20 ± 2.56°) after treatment. A clockwise rotation of the maxilla was more expressed in patients with bone anchors (0.90 ± 2.24°) than in patients with facemask (-0.64 ± 1.88°). The clockwise rotation of jaws increases the ANB angle as well. Clockwise rotation of the mandibula was less in patients with bone anchors (-1.01 ± 2.48°) than in patients with facemask (1.05 ± 1.51°). Divergency of the maxilla and the mandible was less in patients with bone anchors (-2.10 ± 3.73°) than in patients with facemask (1.68 ± 1.77°). Mandibular angle change was almost the same in patients with bone anchors (0.41 ± 4.00°) and in patients with facemask (0.40 ± 2.80°) resulting in a horizontal growth pattern in patients with bone anchors and in a vertical growth pattern in patients with facemask. Forward movement of upper incisors was less in patients with bone anchors (1.69 ± 5.62°) than in patients with facemask (3.35 ± 6.18°). Forward movement of lower incisors was greater in patients with bone anchors (2.03 ± 4.81°) than in patients with facemask (-4.73 ± 5.28°), since the chin cap part of the facemask influences the lower incisor inclination in terms of backward movement of the incisors. Movement of the incisors influences the anterior region of the upper and lower jaw and influences ANB angle as well. Nasolabial angle got larger in patients with bone anchors (4.92 ± 17.59°) than in patients with facemask (-2.58 ± 9.47°), mainly because the patients with bone anchors presented greater initial proclination of the upper incisors already prior to treatment. The change of the occlusal plane inclination was only significant in patients with facemask (2.63 ± 2.43°), whereas it was almost indistinguishable in patients with bone anchors (1.00 ± 3.05°). Wits was smaller but improved more during treatment in patients with bone anchors (2.01 ± 2.65 mm) than in patients with facemask (1.85 ± 4.09 mm), depending on the changes of the maxilla and the mandibula and incisor inclination of both jaws.

The forward movement of the upper lip was less in patients with bone anchors (-0.58 ± 2.07 mm) than in patients with facemask (0.95 ± 1.90 mm), depending on the movement of the maxilla and upper incisors. Backward movement of the lower lip was less in patients with bone anchors (-1.42 ± 2.02 mm) than in patients with facemask (0.42 ± 1.39 mm), depending on the movement of the mandibula and lower incisors.

All patients with a facemask presented a change of the deciduous to the permanent upper and lower incisors during treatment. This change influenced upper and lower incisor inclination as well. Due to the age discrepancy of patients with facemask and with bone anchors, a clear comparison of the two maxillary protraction protocols is limited, but nevertheless of significant clinical interest, since patients requiring treatment for a skeletal class III are referred to the orthodontist at different ages. The ideal control for both groups of our study would consist of untreated growing class III patients with corresponding age. However, the ALARA principle prohibits X-rays in patients without appropriate treatment. Our study comprised cephalometric radiographs in patients with immediate treatment need. Apart from this, growing patients with a skeletal class III for whom treatment is indicated should not be left untreated for ethical reasons.

Finally, certain clinical aspects of the two treatment approaches of our study must be considered. The extraoral facemask is more bulky and less tolerated than intraoral bone anchors and class III elastics. The amount of facemask use per day is smaller than for bone anchors. Two surgical procedures are required for the bone anchors, that is, the insertion and removal of the miniplates. After protraction of the upper jaw, however, the bone anchors can be used for anchoring or distalisation during subsequent orthodontic treatment. In addition, bone anchors are a useful treatment approach for patients whose facemask therapy had not been successful, and as an attempt to avoid or at least to decrease the amount of later orthognathic surgery, especially in patients that have been too old for facemask treatment.

## Conclusions

Bone anchors and facemask therapy improves the relationship of the maxilla and mandibula in class III malocclusion patients and leads to favorable outcomes. Skeletal and soft tissue changes were remarkable for the short term for both groups. Unwanted side effects were reduced using bone anchors. Nevertheless, even with bone anchors complications could not be avoided. However, bone anchors are capable of being integrated easily into everyday life, because elastics could be worn even at school. Nonetheless, larger patient numbers are necessary for a final assessment, especially regarding long-term stability and possible later need for orthognathic surgery.

## Data Availability

No datasets were generated or analysed during the current study.
